# Optical Zitterbewegung effect in arrays of helical waveguides

**DOI:** 10.1515/nanoph-2024-0329

**Published:** 2024-09-05

**Authors:** Kaiyun Zhan, Qixuan Chen, Qian Zhang, Tingjun Zhao, Hanqiang Qin, Haolong He, Guangting Yao

**Affiliations:** College of Science, China University of Petroleum (East China), Qingdao 266580, China

**Keywords:** Zitterbewegung effect, helical waveguides, Floquet engineering

## Abstract

Owing to its topological properties and band collapse, Floquet helical photonic lattices have gained increasing attention as a purely classical setting to realize the optical analogues of a wide variety of quantum phenomena. We demonstrate both theoretically and numerically that light propagation in an appropriately designed helical superlattice can exhibit spatial photonic Zitterbewegung effect, i.e., a quiver spatial oscillatory motion of the beam center of mass around its mean trajectory, in both one- and two-dimensional cases. The lattice spacing determines the effective coupling strength between adjacent helical waveguides, and further drastically not only affects the oscillation amplitude and frequency, but also invert their direction of drift when the effective coupling strength is tuned from positive to negative. Complete arrest and inversion of the drift direction of Zitterbewegung effect are reported.

## 1 Introduction

Zitterbewegung (ZB) oscillation represents a high frequency trembling motion of free relativistic Dirac particles described by the Dirac equation, which is caused by the interference between the positive and negative energy states [1], [2]. This phenomenon exhibits an oscillatory motion of a propagating wavepacket transverse to its ballistic trajectory in the absence of external fields. However, a direct experimental observation of ZB effect is difficult owing to its extremely high frequency and small amplitude [2], [3]. Due to the similarity between the relativistic electron and two interacting bands in solid physics, ZB phenomena have so far predicted in a wide variety of quantum and classical physical systems, such as semiconductors [4], [5], superconductors [6], [7], topological insulators [8], Bose–Einstein condensate [9], ultracold atoms [10], plasma [11], as well as optical systems [12], [13], [14], [15], [16], [18], [19]. The experimental observation of quantum phenomena in the electron case is severely hindered by the complex interactions and the limited scale. No such difficulties exist in the optical context, the use of photonic systems to mimic quantum phenomena have attracted continuous and increasing interest from both fundamental and applied viewpoints. More specifically, optical analogs of the relativistic ZB effect have recently been investigated in modulated photonic lattices, which provide a powerful tool for the control of the fundamental aspects of light propagation. In binary waveguide array, the two-component (spinor) wave function dynamics of the Dirac equation can be simulated by paraxial light propagation and the spinor is represented by two interleaved sublattices [12], [13], [14]. In two-dimensional photonic crystals, photonic analogs of the ZB can be observed by measuring the time dependence of the transmission coefficient through photonic crystal slabs [15], [16]. ZB effect is also demonstrated in a honeycomb lattice of coupled rods with slowing varying radii along the direction of propagation, which possesses photonic Weyl points and become Dirac points when projected down to a two-dimensional momentum space [17]. Furthermore, in photonic microcavities, an analogue ZB resulting from the photonic spin–orbit coupling on the real space propagation of polariton wavepackets is also experimentally demonstrated [18]. Another optical simulation of ZB is based on frequency-conversion process of optical pulses in quadratic media, the mismatch of the group velocities of signal and sum-frequency fields leads to an oscillating motion [19].

As mentioned above, optical ZB are most frequently considered in periodic structures with constant transverse modulation by changing the waveguide strength and spacing or the background refractive index. Nevertheless, the periodically driving-induced behaviors of light in space–time periodic optical system can be understood by directly inspecting the band structure in the Floquet picture [20], [21]. Floquet engineering provides additional possibilities for control of beam propagation [22]. Among them, photonic lattice composed of evanescently coupled single mode helical waveguides, fabricated using femtosecond direct laser writing, has received considerable attention for its topological properties and band collapse. The optical analogues of some quantum phenomena originally predicted for electrons have been realized in array of helical waveguides, including Bloch oscillations [23], [24], Zener tunneling [25], conical diffraction and dynamic localization [26]. However, the study of the coexistence and interplay between the different modes remains largely unexplored in the helical waveguide arrays. It is interesting to investigate the interplay between the Floquet mechanism and the inner state interference.

In this work, we show both theoretically and numerically that the optical ZB effect can be realized in Floquet photonic lattices composed of coupled helical waveguides. By employing an alternation of distances between adjacent waveguides, the quasi-energy bands of the superlattice can be effectively controlled, complete collapse and band curvature inversion are obtained. That affects the amplitude, frequency and direction of ZB effect. Even they can be completely arrested or their direction can be reversed. ZB-like motion with the lateral shift is also demonstrated in two-dimensional Floquet photonic lattices composed of coupled vertical and horizontal stacks of Su-Schrieffer-Heeger (SSH) [27] chains. Both completely arrest and inversion of lateral shift are also observed.

## 2 Optical ZB effect in one-dimensional helical waveguides

**Figure 1:**
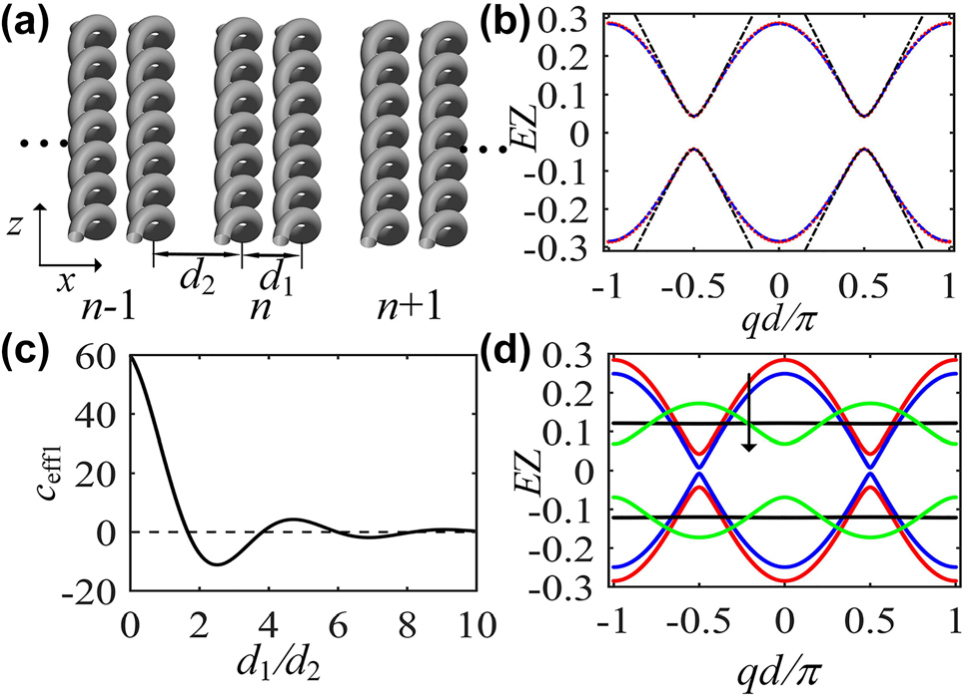
The quasi-energy spectrum of one-dimensional array of helical waveguides. (a) Schematic of one-dimensional array of helical waveguides. Each dimer is distinguished by the index *n*. (b) Floquet quasi-energy spectrum (red curve) and dispersion relation obtained by the analytic treatment (blue curve) at *d*_1_/*d*_2_ = 0.8, black dotted curves show the corresponding hyperbolic dispersion curves. (c) Effective coupling constant *c*_eff1_ as a function of the value of *d*_1_/*d*_2_. (d) Dispersion relations of the lattice with *d*_1_/*d*_2_ = 0.8 (red curve), 1, 1.66 (blue curve) and 2 (green curve). Other parameters are *Z* = 0.5 cm, *R* = 8 μm, *n*_0_ = 1.45, *d*_2_ = 10 μm, *λ* = 633 nm, *c*_0_ = 60 m^−1^ and *b* = 0.03.

An example of scheme of one-dimensional setup is shown in Figure 1(a), which is somewhat similar to SSH lattice. A period array composed of coupled dimers. *d*_1_ is the spacing between sites inside dimers, and *d*_2_ is the spacing between two neighboured dimers. The governing equation, for the complex amplitude of the field *ψ*(*x*, *y*, *z*) of a light beam propagating in a helical waveguide array with the helix spatial period *Z* and radius *R*, can be derived from the Maxwell equations. By using the slowly amplitude approximation, the following two-dimensional Schrödinger-type equation can be arrived:

(1) $$i\frac{\partial \psi }{\partial z}+\frac{1}{2k_{0}}\left(\frac{\partial^{2}}{\partial x^{2}}+\frac{\partial^{2}}{\partial y^{2}}\right)\psi +\frac{k_{0}}{n_{0}}\Delta n\left(x,y,z\right)\psi =0,$$

where *ψ*(*x*, *y*, *z*) is the envelope of the electric field *E*(*x*, *y*, *z*) = *ψ*(*x*, *y*, *z*)exp(*ik*_0_ *z* − *iω*_0_ *t*), *k*_0_ = 2*πn*_0_/*λ* is the wavenumber, *λ* is the wavelength, *n*_0_ is the background refractive index, *ω*_0_ = 2*πc*_*v*_/*λ* is the frequency with *c*_*v*_ being the light speed in the vacuum, Δ*n*(*x*, *y*, *z*) is the refractive change that define the helical waveguides. To change the *z*-dependency of the waveguide system into a stationary and straight one, we do a coordinate transform operation through the relations *x*′ = *x* + *R* cos(Ω*z*), *y*′ = *y* + *R* sin(Ω*z*) and *z*′ = *z*, where Ω = 2*π*/*Z* is longitudinal frequency of the helix corresponding to periodicity *Z*. Now the Eq. (1) is written as [21], [24], [28]:

(2) $$\begin{array}{ll} & i\frac{\partial \psi^{\prime }}{\partial z^{\prime }}+\frac{1}{2k_{0}}{\left(▽+iA\left(z^{\prime }\right)\right)}^{2}\psi^{\prime }+\frac{k_{0}R^{2}\Omega^{2}}{2}\psi^{\prime }\\ & +\frac{k_{0}}{n_{0}}\Delta n\left(x^{\prime },y^{\prime },z^{\prime }\right)\psi^{\prime }=0 \end{array}$$

where *ψ*′ = *ψ*′(*x*′, *y*′, *z*′), **▽** = (∂*x*′, ∂*y*′); **A**(*z*′) = *A*_0_[sin(Ω*z*′), −cos(Ω*z*′), 0] is the artificial vector potential induced by the helicity of the waveguides with amplitude *A*_0_ = *k*_0_ *R*Ω. The propagation of light in such weakly coupled waveguides with the nearest-neighbor interaction can be described by the tight-binding model:

(3) $$\begin{array}{cc} i\frac{\partial \psi_{2n}}{\partial z} & =-c\left(d_{1}\right){\text{e}}^{\text{i}\left[A\left(z\right)\cdot r_{2n+1,2n}\right]}\psi_{2n+1}\\ & \;-c\left(d_{2}\right){\text{e}}^{\text{i}\left[A\left(z\right)\cdot r_{2n-1,2n}\right]}\psi_{2n-1},\\ i\frac{\partial \psi_{2n+1}}{\partial z} & =-c\left(d_{1}\right){\text{e}}^{\text{i}\left[A\left(z\right)\cdot r_{2n,2n+1}\right]}\psi_{2n}\\ & \;-c\left(d_{2}\right){\text{e}}^{\text{i}\left[A\left(z\right)\cdot r_{2n+2,2n+1}\right]}\psi_{2n+2}, \end{array}$$

To simplify notation, we have omitted the superscript ′. *ψ*_2*n*_ and *ψ*_2*n*+1_ denote the amplitude functions linked with left and right waveguides inside dimers. **r** is the displacement vector between the neighboring waveguides, *c*(*d*_1,2_) is the coupling strength, and assumed to be decay exponentially with the distance *c* = *c*_0_ exp(−*bd*) [29], [30], where *b* characters the exponential decay rate.

It is instructive to analyze the band structure of one-dimensional helical waveguides. Due to the periodicity of the Hamiltonian *H*(*q*_*x*_, *z*) = *H*(*q*_*x*_, *z* + *Z*) [25] where *q*_*x*_ is transverse Bloch momentum, Floquet theory can be applied to drive a band structure of so-called quasi-energies *E*. A solution of such *z*-dependent coupled mode equations is the Floquet states *ψ*_*n*_(*z*) = *ϕ*_*n*_(*z*) exp(*iEz* + *iq*_*x*_ *nd*) with *d* = *d*_1_ + *d*_2_ and *ϕ*_*n*_(*z*) = *ϕ*_*n*_(*z* + *Z*). The quasi-energies can be calculated directly by $E\left(q_{x}\right)=\frac{i}{Z}\log(\exp\left(-i\int_{0}^{Z}H\left(q_{x},z\right)dz\right)$ . A graphic presentation of the Floquet quasi-energy spectrum is shown in Figure 1(b). Two minibands are formed, which intersect at the edges of the Brillouin zone for *d*_1_ = *d*_2_, whereas the two minibands are well separated energetically when *c*(*d*_1_) ≠ *c*(*d*_2_). It is shown that, near the Brillouin zone edge, the dispersion relations approximate two opposite hyperbolas, as shown in Figure 1(b), which is similar to that for positive-energy and negative-energy branches of a freely moving relativistic massive particle, therefore beam propagation at incidence angles near the Bragg angle can mimic the temporal dynamic of a one-dimensional massive Dirac electron, that is to say, our model enables an easy visualization in space of the photonic ZB phenomenon.

To verify the above analysis, an analytic treatment of the light dynamics is developed, which can also provide some valuable insight. Discrete diffraction in arrays of helical waveguides occurs like in a straight array with an effective coupling after every period, which can be computed using averaging methods and is given by *c*_eff1_ = *c*(*d*_1_)*J*_0_(*k*_0_ *R*Ω*d*_1_) and *c*_eff2_ = *c*(*d*_2_)*J*_0_(*k*_0_ *R*Ω*d*_2_) for the spacing between adjacent lattice sites *d*_1_ and *d*_2_, respectively [24], where *J*_0_ is the Bessel function of the first kind. Coupled-mode equations then take the following simplified form:

(4) $$\begin{array}{c} i\frac{\partial \psi_{2n}}{\partial z}+c_{\text{eff}1}\psi_{2n+1}+c_{\text{eff}2}\psi_{2n-1}=0,\\ i\frac{\partial \psi_{2n+1}}{\partial z}+c_{\text{eff}1}\psi_{2n}+c_{\text{eff}2}\psi_{2n+2}=0, \end{array}$$

The corresponding band structure reads

(5) $$E\left(q_{x}\right)=\pm \sqrt{{\left(c_{\text{eff}1}-c_{\text{eff}2}\right)}^{2}+4c_{\text{eff}1}c_{\text{eff}2}\cos^{2}\left(dq_{x}/2\right)},$$

which is entirely consistent with Floquet quasi-energy spectrum of Figure 1(b) (the red and blue curves coincide). Let us now analyze the spectrum of the photonic lattice in detail. Equation (5) indicates that the effective couplings can be tuned from positive to negative by varying the waveguide separation, and flat band appears at *d*_1cr_ = 1.66*d*_2_, corresponding to zero of *c*_eff1_, as shown in Figure 1(c), the band structure experiences a complete band collapse in the first Brillouin zone (see Figure 1(d)) and would support compact localized modes. A further increase of the distance *d*_1_, results in inversion of the respective dispersion curves. Obviously, the effect of band collapse would be repeat as *d*_1_ increases. Around |*q*_*x*_| = *π*/*d*, the two minibands are separated by a gap *β* = 2|*c*_eff1_ − *c*_eff2_|, and exhibit two opposite hyperbolas $\epsilon =\pm \sqrt{{\left(c_{\text{eff}1}-c_{\text{eff}2}\right)}^{2}+c_{\text{eff}1}c_{\text{eff}2}Q^{2}}$ (see Figure 1(b)), where *Q* = *dq*_*x*_ − *π*. Let us assume that the array is excited by a broad beam *G*(*x*) tilted at the Bragg angle, the modes are excited with a nearly equal amplitude, but the phase difference between adjacent waveguides is equal to *π*/2 across the array. By setting *ψ*_2*n*_(*z*) = (−1)^*n*^ *φ*_1_(*n*, *z*), *ψ*_2*n*−1_(*z*) = −*i*(−1)^*n*^ *φ*_2_(*n*, *z*) and introducing the continuous transverse coordinate *ξ* ↔ *n* = *x*/*d*, the two-component spinor $\varphi \left(\xi ,z\right)={\left(\varphi_{1},\varphi_{2}\right)}^{T}$ satisfies the following equation:

(6) $$i\frac{\partial \varphi }{\partial z}=-ic_{\text{eff}1}\sigma_{x}\frac{\partial \varphi }{\partial \xi }+\left(c_{\text{eff}1}-c_{\text{eff}2}\right)\sigma_{y}\varphi ,$$

where *σ*_*x*,*y*_ are the Pauli matrices. Equation (6) corresponds to the one-dimensional Dirac equation for an electron of mass *m*_0_ provided that the formal change *c*_eff1_ ↔ *c*_*v*_, $\left(c_{\text{eff}1}-c_{\text{eff}2}\right)↔m_{0}c_{v}^{2}/\hbar$ , *z* ↔ *t* is made, where *ℏ* is the reduced Planck constant. Obviously, the Dirac equation in its discretized form can be mapped on an array of helical waveguides with periodically alternating transverse lattice spacing. The Dirac spinor wave function can also be mapped onto the spatial evolution of the field amplitudes *φ*_1_ and *φ*_2_. As a result, ZB effect is observed as a quivering spatial oscillatory motion of the beam center of mass. By solving this equation in the momentum *k* space, one gets:

(7) $$\begin{array}{cc} {\hat{\varphi }}_{1}\left(k,z\right) & =\hat{G}\left(k\right)\left[\cos\left(\epsilon z\right)\right]-ic_{\text{eff}1}k\frac{\sin\left(\epsilon z\right)}{\epsilon }\\ & \;-\left(c_{\text{eff}1}-c_{\text{eff}2}\right)\frac{\sin\left(\epsilon z\right)}{\epsilon },\\ {\hat{\varphi }}_{2}\left(k,z\right) & =\hat{G}\left(k\right)\left[\cos\left(\epsilon z\right)\right]-ic_{\text{eff}1}k\frac{\sin\left(\epsilon z\right)}{\epsilon }\\ & \;+\left(c_{\text{eff}1}-c_{\text{eff}2}\right)\frac{\sin\left(\epsilon z\right)}{\epsilon }, \end{array}$$

where ${\hat{\varphi }}_{1,2}\left(k,z\right)=\int dk\varphi_{1,2}\left(\xi ,z\right)\exp\left(-ik\xi \right),\hat{G}\left(k\right)=\int d\xi \hat{G}\left(\xi \right)\exp\left(-ik\xi \right)$ . The expectation value of position for the relativistic particle is defined by $\left\langle \xi \right\rangle \left(z\right)=i\int dk\left[{\hat{\varphi }}_{1}^{*}\left(k\right)\partial_{k}{\hat{\varphi }}_{1}\left(k\right)+{\hat{\varphi }}_{2}^{*}\left(k\right)\partial_{k}{\hat{\varphi }}_{2}\left(k\right)\right]$ , which can be used to determine the spatial motion of the beam center through the relation ⟨*n*⟩(*z*) ≃ 2⟨*ξ*⟩(*z*) + 1/2. ⟨*ξ*⟩(*z*) is calculated as

(8) $$\begin{array}{ll} 〈\xi 〉\left(z\right) & =〈\xi 〉\left(0\right)+v_{0}z+2\pi c_{\text{eff}1}{\left(c_{\text{eff}1}-c_{\text{eff}2}\right)}^{2}\\ & \;\times \int dk\frac{\sin\left(2\epsilon z\right)|\hat{G}\left(k\right){|}^{2}}{\epsilon^{3}}, \end{array}$$

where $v_{0}=4\pi c_{\text{eff}1}^{3}\int dkk^{2}|\hat{G}\left(k\right){|}^{2}/\epsilon^{2}$ .

The main result of our analysis is summarized by dividing the evolution of the expectation value of the position into the drift and ZB component. The third term denotes the ZB oscillation. The frequency of ZB, which can be estimated as *ω* = 2|*ϵ*| = 2|*c*_eff2_ − *c*_eff1_|, grows linearly with increasing a gap *β* = *ω*. It can be seen clearly that the motion of the wave packet is accompanied by a transverse drift at a constant velocity *v*_0_ and the direction of drift depends on the polarity of *c*_eff1_, which can be effectively regulated through properly adjusting the lattice distance *d*_1_.

**Figure 2:**
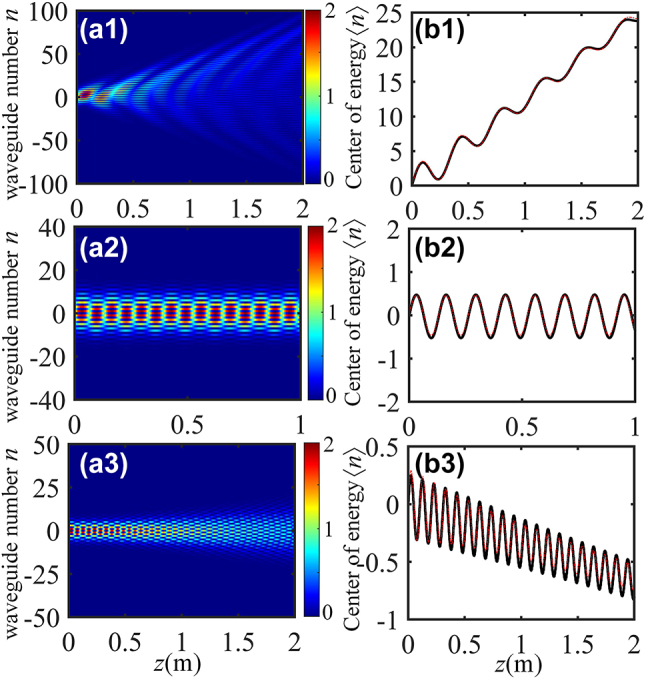
Optical ZB supported by one-dimensional array of helical waveguides with *d*_1_/*d*_2_ = 0.8, 1.66 and 2, respectively (from top to bottom). Left and right panels show the beam propagation and the evolution of center of energy, respectively. The black solid and red dotted curves correspond to the direct numerical simulated of Eq. (3) and analytical results based on Eq. (8), respectively.

Now, going back to one-dimensional helical waveguides (Figure 1(a)), and examining the mode interference of the system. In order to reduce the influence of the boundary effect, the model consists of more than 400 dimers. The wave transmission for a broad Gaussian beam excitation $\left[\psi_{2n}\left(0\right),\psi_{2n+1}\left(0\right)\right]=\left(i^{n},i^{n+1}\right)\exp\left[-{\left(nd/w_{0}\right)}^{2}\right]$ (corresponding to Bragg angle *λ*/(2*dn*_0_)) is investigated by directly solving Eq. (3), where *w*_0_ = 10*d*. Beam dynamics reveals that a clear trembling motion of the beam – corresponding to the optical ZB oscillation – is observed, as shown in Figure 2(a1). One immediately notes the damping of ZB oscillations is also visible, which is ascribable to the angular spectral broadening of the launching broad Gaussian beam. The corresponding oscillation period along the propagation is *T* = 0.64 m illustrated in Figure 2(b1), which is in good agreement with the theoretical predictions (*T* = 2*π*/*β*). The influence of *d*_1_ on the effective coupling strength *c*_eff1_ is summarized in Figure 1(c). With increasing the distance *d*_1_ (*d*_1_ > *d*_2_), the gap is extended, and the oscillation period increases and the amplitude decreases. In fact, the initial oscillation amplitude can be estimated in the limit at *k* = 0. Based on Eq. (8), the frequency and amplitude of ZB effect are given by *ω*_ZB_ = 2|*c*_eff1_ − *c*_eff2_| and *R*_ZB_ = *πc*_eff1_/*ω*_ZB_, respectively. The analytical and numerical results agree with each other very well.

The lattice spacing affects not only amplitude and frequency of the optical ZB, but it can also arrest this effect and even invert their direction of drift. When the intracell spacing *d*_1_ is small, oscillations occur in the region *n* > 0, the lateral shift of the center of beam energy decreases with increase of *d*_1_. At *d*_1_ = 1.66*d*_2_, corresponding to the special case *c*_eff1_ = 0, two flat band pinned at *E* = ±*c*_eff2_ are presented. This means that excitation in such a lattice will propagate without diffraction, which results in complete localization of the wavepacket in the system. This effect is known as dynamic localization. The ZB oscillations are undamped and they are localized, as shown in Figure 2(a2) and (b2). Further increasing the distance *d*_1_, corresponding to negative *c*_eff1_, subsequent reappearance of ZB effect at *d*_1_ > *d*_*1cr*_ is accompanied by the reversal of the direction of oscillations, where ZB effect appears in the region *n* < 0 (see Figure 2(a3) and (b3)).

**Figure 3:**
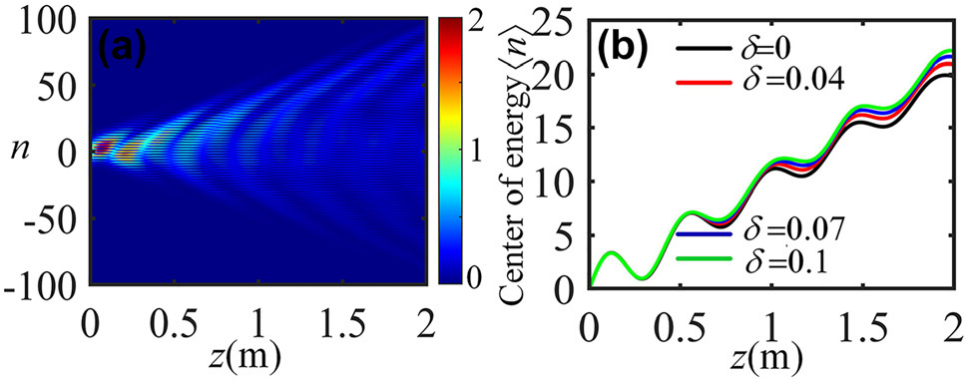
Robustness of optical ZB with *d*_1_/*d*_2_ = 0.8 under the random perturbation. (a) Light evolution at *δ* = 0.1. (b) Center of the energy with different perturbation.

We further investigate the robustness of the optical ZB in arrays of helical waveguides, by introducing the random noise in the coupling strength *c*(*d*_1,2_). A coefficient (1 + *δr*_*n*_) is multiplied on coupling strength, where *δ* is the perturbation strength, *r*_*n*_ is a random number between −1 and 1. Figure 3 shows that, the beams exhibit the similar ZB-like lateral shifts with *δ* up to 10 % (namely, *δ* = 0.1). Consequently, they show a great robustness against perturbation of the coupling strength.

**Figure 4:**
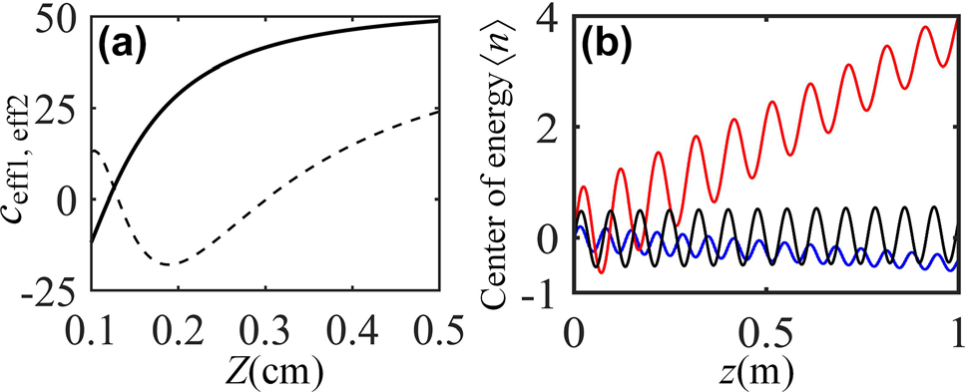
Optical ZB for different helix spatial periods. (a) Effective coupling constants *c*_eff1_ (solid curve) and *c*_eff2_ (dotted curve) as a function of the helix spatial period *Z* with *d*_1_/*d*_2_ = 0.4. (b) Lateral shifts of the center of energy at *Z* = 0.40 cm (red curve), 0.31 cm (black curve) and 0.23 cm (blue curve). Other parameters are same as that in Figure 1.

Here, we focus on the impact of lattice spacing on the ZB effect in arrays of helical waveguides, it is shown that longitudinal rotation of waveguides (helix radius and period) leads to notable variations of Floquet quasi-energy spectrum, that will certainly drastically affect the ZB oscillations. Figure 4(a) shows the effective coupling constants as a function of the helix spatial period. The ZB oscillations can occur in the regions *n* > 0 and *n* < 0 at *Z* = 0.40 cm and 0.23 cm, respectively. ZB effect with undamped amplitude is also observed at *Z* = 0.31 cm.

## 3 Optical ZB effect in two-dimensional helical waveguides

**Figure 5:**
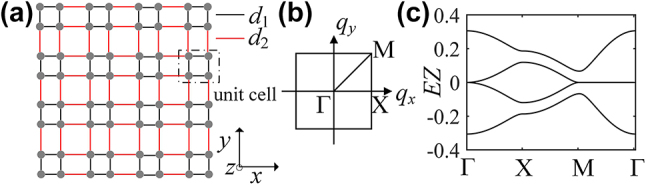
Dispersion relation of two-dimensional helical waveguide array. (a) Schematic of two-dimensional helical waveguide array. (b) Corresponding first Brillouin zone of the square lattice in (a), (c) dispersion relation of two-dimensional model with *d*_1_/*d*_2_ = 0.8 along high symmetry points. Other parameters are same as Figure 1.

The ZB-like motion is further investigated in two-dimensional geometry shown in Figure 5(a), which is based on two-dimensional SSH model composed of coupled vertical and horizontal stacks of SSH chains [31]. (*m*, *n*) are the integers that enumerate the lattice sites. The lattice consists of four sites per unit cell with dimerized nearest neighbor couplings. Light propagation can be described by the following coupled-mode equation for the modal field amplitudes *ψ*_2*n*,2*m*_,

(9) $$\begin{array}{c} i\frac{\partial \psi_{2n,2m}}{\partial z}+c_{\text{eff}1}\psi_{2n+1,2m}+c_{\text{eff}2}\psi_{2n-1,2m}\\ +c_{\text{eff}1}\psi_{2n,2m+1}+c_{\text{eff}2}\psi_{2n,2m-1}=0. \end{array}$$

After setting *ψ*_2*n*,2*m*_ = (−1)^*n*+*m*^ *φ*_1_(*n*, *m*), *ψ*_2*n*−1,2*m*_ = −*i*(−1)^*n*+*m*^ *φ*_2_(*n*, *m*), *ψ*_2*n*,2*m*−1_ = −*i*(−1)^*n*+*m*^ *φ*_3_(*n*, *m*) and *ψ*_2*n*−1,2*m*−1_ = (−1)^*n*+*m*^ *φ*_4_(*n*, *m*), and introducing the two continuous transverse coordinates *η* ↔ *n*, *ζ* ↔ *m*, the four-component bispinor $\varphi \left(\eta ,\zeta ,z\right)={\left(\varphi_{1},\varphi_{2},\varphi_{3},\varphi_{4}\right)}^{T}$ satisfies the following two-dimensional Dirac-type equation:

(10) $$i\frac{\partial \varphi }{\partial z}=-ic_{\text{eff}1}\left(\alpha \frac{\partial \varphi }{\partial \eta }+\rho \frac{\partial \varphi }{\partial \zeta }\right)-i\left(c_{\text{eff}1}-c_{\text{eff}2}\right)\tau \varphi ,$$

where, *α*, *ρ* and *τ* are 4 × 4 matrices with all elements equal to zero, except *α*_1,2_ = *α*_2,1_ = *α*_3,4_ = *α*_4,3_ = 1, *ρ*_1,3_ = *ρ*_2,4_ = *ρ*_3,1_ = *ρ*_4,2_ = 1 and *τ*_1,2_ = *τ*_1,3_ = −*τ*_2,1_ = *τ*_2,4_ = −*τ*_3,1_ = *τ*_3,4_ = −*τ*_4,2_ = −*τ*_4,3_ = 1. Correspondingly, two-dimensional optical ZB effect can also be realized in arrays of helical waveguides.

**Figure 6:**
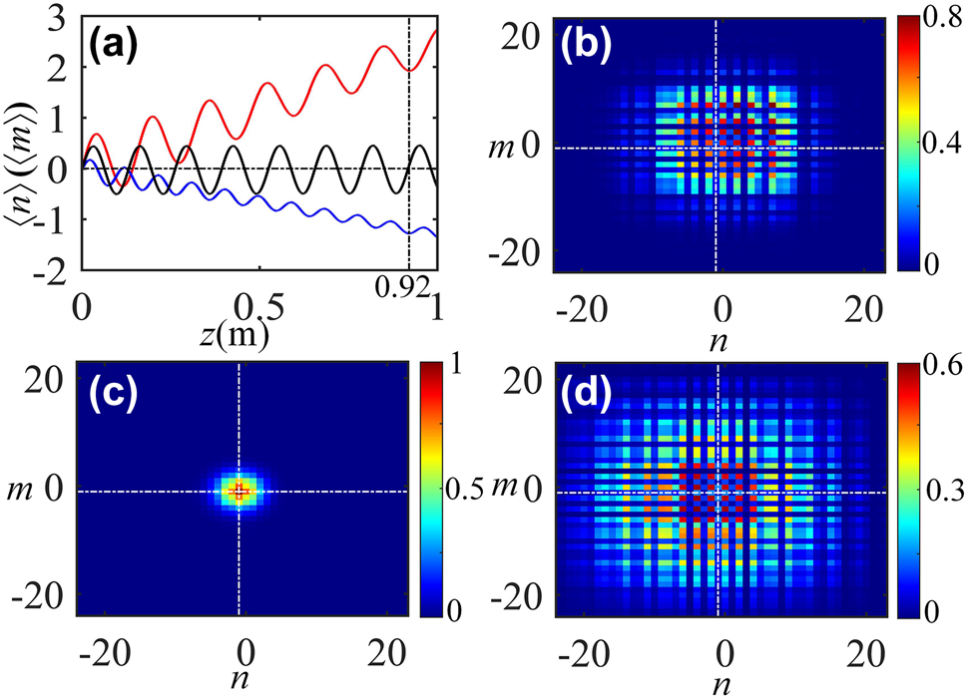
Optical ZB effect in two-dimensional helical waveguides. (a) Lateral shifts of the center of the Gaussian beam in two-dimensional geometries at *d*_1_/*d*_2_ = 1.5 (red line), 1.66 (black line) and 2.2 (blue line). (b)–(d) Field modulus distributions at *z* = 0.92 m for *d*_1_/*d*_2_ = 1.5, 1.66 and 2.2, respectively.

Substituting the periodic solution *ψ*_*n*,*m*_(*z*) = *ϕ*_*n*_(*z*) exp[*iEz* + *i*(*q*_*x*_ *n* + *q*_*y*_ *m*)*d*] in the form of Floquet–Bloch-like wave in Eq. (3), where *q*_*x*_ and *q*_*y*_ are the wave numbers along *n* and *m*, the dispersion relation of the two-dimensional helical waveguide array along high symmetric points (Figure 5(b)) is shown in Figure 5(c). At the high symmetry point M, there are three eigenvalues *E*_1_ = −*E*_3_ = 2|*c*_eff2_ − *c*_eff1_|, and *E*_2_ = 0. Similar to the case of one-dimensional geometry, the waveguide array is also excited by a broad circular Gaussian beam, $\psi_{n,m}\left(0\right)=i^{n+m}\exp\left[-{\left(nd/w_{0}\right)}^{2}-{\left(md/w_{0}\right)}^{2}\right]$ with *w*_0_ = 6*d*. Figure 6 shows the lateral ZB-like motion of the Gaussian beam. Although there contain two oscillation frequencies generated by interferences between the upper and zero modes (*E*_1_ − *E*_2_), and between the upper and low modes (*E*_1_ − *E*_3_). The former plays the leading role in determined the ZB-like motion. The oscillation period is determined by the *T* = 2*π*/*E*_3_. Figure 6(a) show the lateral shifts of the Gaussian beams with *d*_1_/*d*_2_ = 1.5, 1.66 and 2.2. At *c*_eff1_ = 0 (*d*_1_/*d*_2_ = 1.66), corresponding to dynamic localization, the beams oscillation without lateral shift shown in Figure 6(c). When *d*_1_/*d*_2_ = 1.5 (for *c*_eff1_ > 0), the wave packet expands in both transverse dimensions, optical ZB-like motion occurs with the lateral shift along the bisectrix of the (*n*, *m*) plane in the first quadrant(*n*, *m*) > 0, as shown in Figure 6(b) The inversion of the direction of this optical ZB-like motion is also confirmed with the lateral shifts in the third quadrant (*n*, *m*) < 0 when *d*_1_/*d*_2_ = 2.2 (for *c*_eff1_ < 0), as shown in Figure 6(d). The lateral shifts of the two beams are opposite shown in Figure 6(a). The corresponding oscillation periods are *T* = 0.15 m, 0.13 m and 0.10 m, respectively, which are agreement with the theoretical predicated.

## 4 Conclusions

In conclusion, propagation of light waves in one-dimensional and two-dimensional Floquet photonic system made of SSH-like helical arrays of evanescently coupled optical waveguides is theoretically and numerically investigated and shown to provide a classic wave optics analog of the ZB effect, where a spatial oscillatory motion of an optical beam is observed. In one-dimensional case, Floquet quasi-energy spectrum consists of two minibands. Near the edges of the first Brillouin zone, the dispersion curve looks like that for positive-energy and negative-energy branches of a freely moving relativistic massive particle and light transport simulates the temporal dynamics (ZB effect) of the relativistic Dirac equation. The lattice spacing determines the effective coupling strength, and further drastically not only affects the oscillation amplitude and frequency, but also invert their direction of drift when the effective coupling strength is tuned from positive to negative. The ZB-like motion is also demonstrated in two-dimensional geometry. At the high symmetric point M, there are three eigenvalues, the interference between the upper and zero modes plays the leading role in determined the ZB-like motion. Their oscillation and direction strongly depend on waveguide spacing (coupling strength). Complete arrest and inversion of the drift direction of ZB effect are reported. Robustness of the optical ZB phenomenon to the perturbations of coupling strengths is also demonstrated. Here, we focus on the impact of lattice spacing on the ZB effect in arrays of helical waveguides, it is shown that longitudinal rotation of waveguides (helix radius and period) leads to notable variations of Floquet quasi-energy spectrum, that can certainly drastically affect the ZB oscillations. Floquet engineering provides additional possibilities for control of ZB effect. Our results will not only enrich the ZB research in Floquet waveguide arrays but also provide a new opportunity to manipulate light propagation. Furthermore, due to its easy accessibility and high controllability, we hope the Floquet photonic lattices composed of coupled helical waveguides enables an easy visualization of other optical analogues of relativistic quantum phenomena (such as Klein tunneling).

## References

[1] Huang K. (2012). On the Zitterbewegung of the Dirac electron. *Am. J. Phys.*.

[2] Krekora P., Su Q., Grobe R. (2004). Relativistic electron localization and the lack of Zitterbewegung. *Phys. Rev. Lett.*.

[3] Barut A. O., Bracken A. J. (1981). Zitterbewegung and the internal geometry of the electron. *Phys. Rev. D*.

[4] Zawadzki W., Rusin T. M. (2011). Zitterbewegung (trembling motion) of electrons in semiconductors: a review. *J. Phys.: Condens. Matter*.

[5] Wlodek Z. (2005). Zitterbewegung and its effects on electrons in semiconductors. *Phys. Rev. B*.

[6] Cserti J., Dávid G. (2006). Unified description of Zitterbewegung for spintronic, graphene, and superconducting systems. *Phys. Rev. B*.

[7] Zhang Q., Gong J. (2016). Perfect Zitterbewegung oscillations in the Kitaev chain system. *Phys. Rev. A*.

[8] Shi L., Zhang S., Chang K. (2013). Anomalous electron trajectory in topological insulators. *Phys. Rev. B*.

[9] LeBlanc L. J. (2013). Direct observation of zitterbewegung in a Bose–Einstein condensate. *New J. Phys.*.

[10] Vaishnav J. Y., Clark C. W. (2008). Observing Zitterbewegung with ultracold atoms. *Phys. Rev. Lett.*.

[11] Fan Y., Wang B., Huang H., Wang K., Long H., Lu P. (2015). Plasmonic Zitterbewegung in binary graphene sheet arrays. *Opt. Lett.*.

[12] Longhi S. (2010). Photonic analog of Zitterbewegung in binary waveguide arrays. *Opt. Lett.*.

[13] Dreisow F. (2010). Classical simulation of relativistic Zitterbewegung in photonic lattices. *Phys. Rev. Lett.*.

[14] Longhi S. (2011). Classical simulation of relativistic quantum mechanics in periodic optical structures. *Appl. Phys. B*.

[15] Zhang X. (2008). Observing Zitterbewegung for photons near the Dirac point of a two-dimensional photonic crystal. *Phys. Rev. Lett.*.

[16] Liu X. (2021). Wavepacket self-rotation and helical Zitterbewegung in symmetry-broken honeycomb lattices. *Laser Photonics Rev.*.

[17] Ye W., Liu Y., Liu J., Horsley S. A. R., Wen S., Zhang S. (2019). Photonic Hall effect and helical Zitterbewegung in a synthetic Weyl system. *Light Sci. Appl.*.

[18] Lovett S. (2023). Observation of Zitterbewegung in photonic microcavities. *Light Sci. Appl.*.

[19] Longhi S. (2010). Zitterbewegung of optical pulses in nonlinear frequency conversion. *J. Phys. B*.

[20] Garanovich I. L., Longhi S., Sukhorukov A. A., Kivshar Y. S. (2012). Light propagation and localization in modulated photonic lattices and waveguides. *Phys. Rep.*.

[21] Rechtsman M. C. (2013). Photonic Floquet topological insulators. *Nature*.

[22] Shan J. (2021). Giant modulation of optical nonlinearity by Floquet engineering. *Nature*.

[23] Longhi S. (2007). Bloch dynamics of light waves in helical optical waveguide arrays. *Phys. Rev. B*.

[24] Zhang W., Zhang X., Kartashov Y. V., Chen X., Ye F. (2018). Bloch oscillations in arrays of helical waveguides. *Phys. Rev. A*.

[25] Yang L., Jie R. (2017). Topological Landau–Zener Bloch oscillations in photonic Floquet lieb lattices. ..

[26] Zeuner J. M. (2012). Optical analogues for massless Dirac particles and conical diffraction in one dimension. *Phys. Rev. Lett.*.

[27] Su W. P., Schrieffer J. R., Heeger A. J. (1979). Solitons in polyacetylene. *Phys. Rev. Lett.*.

[28] Lumer Y. (2019). Light guiding by artificial gauge fields. *Nat. Photonics*.

[29] Cáceres-Aravena G., Real B., Guzmán-Silva D., Amo A., Foa Torres L. E. F., Vicencio R. A. (2022). Experimental observation of edge states in SSH-Stub photonic lattices. *Phys. Rev. Res.*.

[30] Szameit A. (2005). Discrete nonlinear localization in femtosecond laser written waveguides in fused silica. *Opt. Express*.

[31] Jin M., Gao Y., Lin H., He Y. H., Chen M. Y. (2022). Corner states in second-order two-dimensional topological photonic crystals with reversed materials. *Phys. Rev. A*.

